# Pre-synaptic TrkB in basolateral amygdala neurons mediates BDNF signaling transmission in memory extinction

**DOI:** 10.1038/cddis.2017.302

**Published:** 2017-07-27

**Authors:** Yuan Li, Dongdong Wang, Yang Li, Hongxia Chu, Lining Zhang, Ming Hou, Xingyu Jiang, Zheyu Chen, Bo Su, Tao Sun

**Affiliations:** 1 Shandong Provincial Key Laboratory of Immunohematology, Qilu Hospital, Shandong University, Jinan, Shandong 250012, P.R. China; 2 Department of Neurobiology, Shandong Provincial Key Laboratory of Mental Disorders, Shandong University School of Medicine, Jinan, Shandong 250012, P.R. China; 3 Department of Immunology, Shandong University School of Medicine, Jinan, Shandong 250012, P.R. China; 4 School of Life Sciences, Shandong University, Jinan, Shandong 250012, P.R. China; 5 Key Laboratory for Biological Effects of Nanomaterials and Nanosafety, National Center for Nanoscience and Technology, Chinese Academy of Sciences, Beijing 100190, P.R. China

## Abstract

Brain-derived neurotrophic factor (BDNF) and its high affinity receptor, TrkB, play an essential role in memory extinction. Our previous work has shown that JIP3 (JNK interacted protein 3) mediates anterograde axonal transport of TrkB through the direct binding of its coiled-coil domain 1 (CC1) with TrkB. Here, we constructed a fluorescent CC1 and enhanced green fluorescent protein (EGFP) fused protein, CC1-EGFP, and found that CC1-EGFP could specifically interrupt TrkB anterograde axonal transport and its localization at the pre-synaptic site. Consistent with this, TrkB-mediated pre-synaptic vesicle release and retrograde axonal signaling transmission were disrupted by CC1-EGFP. Neuronal expression of CC1-EGFP in the basolateral amygdala (BLA) impaired fear memory extinction. And, it blocked BDNF in the BLA-induced enhancement of TrkB phosphorylation in the infralimbic prefrontal cortex (IL). Together, this study not only suggests that pre-synaptic TrkB in BLA neurons is necessary for memory extinction and contributes to the BDNF signaling transduction from the BLA to IL, but also provides CC1-EGFP as a novel tool to specifically regulate pre-synaptic TrkB expression *in vitro* and *in vivo*.

Extinction of conditioned fear memory is the process of reducing the response to a conditioned stimulus (CS) when an animal is repeatedly exposed to the CS without an unconditioned stimulus (US). The process of memory extinction requires complex networks of brain regions, such as the activity of amygdala, prefrontal cortex, and hippocampus.^1^ Currently, the question of how plasticity is conducted within a defined brain region is unclear. Recent studies have focused on the reciprocal and functional connections between the basolateral amygdala (BLA) and infralimbic prefrontal cortex (IL) during memory extinction and revealed that fear memory extinction depends on the elevated neuronal activity of the BLA and IL.^2, 3, 4^ Brain-derived neurotrophic factor (BDNF), a mediator of synaptic plasticity, plays an essential role in fear memory extinction.^5, 6^ A BDNF gene polymorphism (Val66Met) reducing its secretion has been reported to impair the extinction of fear memory.^7^ However, the mechanism underlying BDNF-regulated memory extinction is still unclear.

As a high affinity receptor for BDNF, TrkB exerts its function by activating several intracellular signaling cascades. BDNF binds pre- or post-synaptic TrkB to regulate neuronal activity, neurotransmitter release and mediate BDNF retrograde endocytosis signaling.^8, 9, 10, 11^ Therefore, the BDNF-induced function in neurons depends on the proper trafficking and surface distribution of TrkB. Overexpression of TrkB.T1 in the BLA impaired conditioned fear memory consolidation and extinction, indicating that TrkB in the BLA plays an essential role in mice memory.^12^ However, as a dominant-negative truncation of TrkB, TrkB.T1 blocks both the pre- and post-synaptic functions of TrkB and is unable to distinguish the functional differences of dendritic and axonal TrkB in BLA neurons. To understand the TrkB-mediated memory extinction, it is important to clearly distinguish the function of pre- and post-synaptic TrkB.

Here, we aimed to study the function of pre-synaptic TrkB of BLA neurons in fear memory extinction. TrkB is known to be generated in the cell body and delivered to axonal terminals through anterograde trafficking in neurons. Previously, we have demonstrated that JIP3 mediates TrkB anterograde axonal transport by directly binding to TrkB with its coiled-coil 1 (CC1) domain.^13^ In this study, we fused the CC1 domain with enhanced green fluorescent protein (EGFP) (CC1-EGFP), which acted as a specific inhibitor of TrkB anterograde axonal transport. We demonstrated that CC1-EGFP blocked the synaptic localization of TrkB, which impaired pre-synaptic TrkB function in primary cultured neurons. Furthermore, we found that the expression of CC1-EGFP in the BLA impaired conditioned fear memory extinction by inhibiting the anterograde transmission of BDNF from the BLA to IL. Our results suggested that pre-synaptic TrkB in BLA neurons is responsible for fear memory extinction and provided a new technique to investigate pre-synaptic TrkB *in vivo*.

## Results

### CC1-EGFP blocked the interaction between TrkB and JIP3

**Figure 1:**
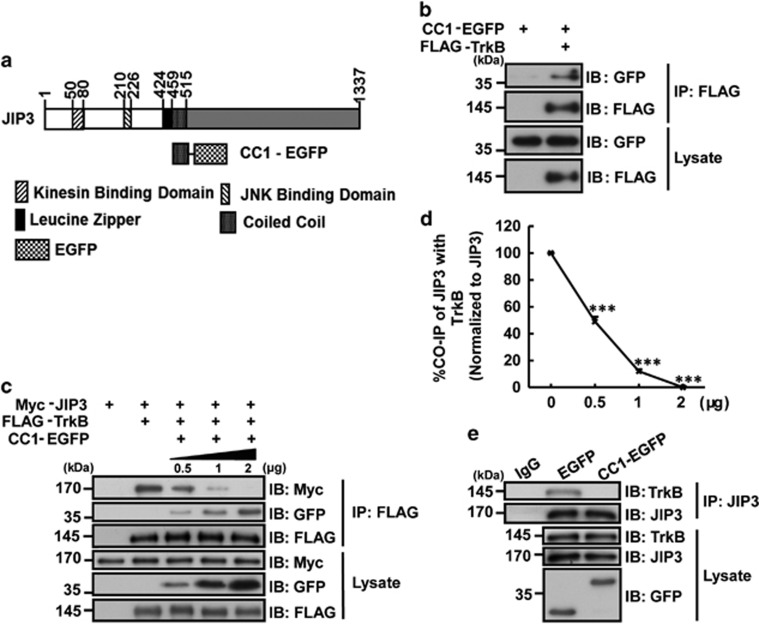
CC1-EGFP interrupted the TrkB-JIP3 interaction. (**a**) Schematic diagram of the domains of the JIP3 protein. CC1 was linked to EGFP to form a novel protein, CC1-EGFP. (**b**) HEK293 cells were co-transfected with FLAG-TrkB and CC1-EGFP. Immunoprecipitation was performed with the anti-FLAG antibody. Immunoblotting was performed with anti-Flag or anti-GFP antibodies, and the results showed that CC1-EGFP interacted with Flag-TrkB. The figure represents three independent experiments that yield similar result. (**c**,**d**) HEK293 cells were co-transfected with Myc-JIP3, FLAG-TrkB and CC1-EGFP. Lysates were immunoprecipitated with the anti-FLAG antibody. Immunoblotting showed that CC1-EGFP blocked the TrkB-JIP3 interaction in a dose-dependent manner. Data are shown as the mean±S.E.M. from three independent experiments (*n*=3, **P<0.01 compared to the Flag-TrkB-positive and CC1-EGFP-negative group). (**e**) CC1-EGFP interrupted the endogenous interaction of JIP3 with TrkB. Neurons infected with EGFP or CC1-EGFP-containing lentivirus were lysed and subjected to immunoprecipitation with an anti-JIP3 antibody. TrkB, JIP3 and GFP were detected by immunoblotting. The figure represents three independent experiments that yield similar result

As shown in Figure 1a, the predicted protein structure of JIP3 has been reported,^14^ and its first coiled-coil (CC1) domain has been verified to directly bind with TrkB.^13^ Therefore, we aimed to use the CC1 domain as a dominant negative peptide to inhibit the binding of TrkB with JIP3 and the expression of TrkB in the pre-synapse. To enhance CC1 peptide stability, we fused it with EGFP to form a CC1-EGFP construct (Figure 1a). Next, we co-transfected Flag-TrkB and CC1-EGFP constructs into HEK293 cells to test whether CC1-EGFP could interact with TrkB. The results showed that after immunoprecipitation with the Flag antibody, CC1-EGFP was found in the immunoprecipitation, suggesting that CC1-EGFP could interact with TrkB (Figure 1b). Then, we found that overexpression of CC1-EGFP significantly decreased the binding of TrkB with JIP3 in a dose-dependent way (Figures 1c and d). To exclude the artifact caused by protein overexpression, we performed an endogenous TrkB/JIP3 co-immunoprecipitation assay in cultured hippocampal neurons, and an endogenous association between TrkB and JIP3 was detected (Figure 1e). More importantly, overexpression of CC1-EGFP significantly inhibited the interaction of TrkB with JIP3 (Figure 1e), which proved that CC1-EGFP could act as a dominant negative for the binding of TrkB and JIP3.

### CC1-EGFP reduced the distribution of TrkB at axonal tips specifically

**Figure 2:**
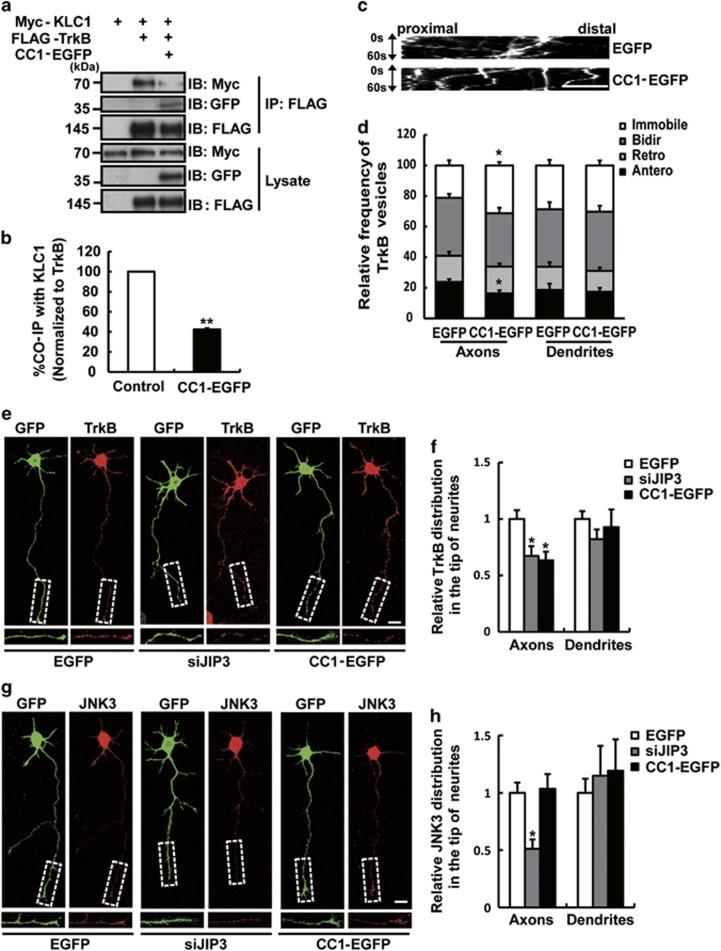
CC1-EGFP blocked TrkB anterograde axonal transport in cultured hippocampal neurons. (**a**,**b**) HEK293 cells were co-transfected with Myc-KLC1, FLAG-TrkB and CC1-EGFP. Lysates were immunoprecipitated with the anti-FLAG antibody. Immunoblotting indicated that CC1-EGFP partially blocked the TrkB-KLC1 interaction. Data are shown as the mean±S.E.M. from three independent experiments (*n*=3, **P<0.01 compared to the Flag-TrkB-positive and CC1-EGFP-negative group). (**c**,**d**) DIV3 hippocampal neurons were co-transfected with GFP or CC1-EGFP and TrkB-mRFP. The kymograph of TrkB-mRFP at the axon was captured every 1 s for 60 s. Scale bar, 20 *μ*m. The relative frequency of retrograde (Retro), anterograde (Antero), bidirectional (Bidir), and immobile TrkB-mRFP puncta at the axons and dendrites was analyzed. The numbers of cells analyzed for each group was>60. Data are shown as the mean±S.E.M. of three independent experiments (*n*=3; *P<0.05 compared to the EGFP group). (**e**,**f**) CC1-EGFP decreased the distribution of endogenous TrkB at the axonal tips. Cultured hippocampal neurons were electroporated with EGFP, siJIP3, or CC1-EGFP, and endogenous TrkB was stained with the anti-TrkB antibody (red) at DIV3. Scale bar, 10 *μ*m. The intensity of TrkB at distal axons and dendrites was analyzed. Data are shown as the mean±S.E.M. from three independent experiments,>60 neurons per experiment (*n*=3; *P<0.05; compared to EGFP group). (**g**,**h**) Neurons were electroporated with EGFP, siJIP3, or CC1-EGFP and were stained for endogenous JNK3 at DIV3. Scale bar, 10 *μ*m. The intensity of endogenous JNK3 at the distal axons and dendrites was analyzed. Data are shown as the mean±S.E.M. of three independent experiments, >60 neurons per experiment (*n*=3; *P<0.05 compared to the EGFP group)

JIP3 plays a key role in kinesin-related TrkB anterograde axonal transport.^15^ To verify whether CC1-EGFP could interrupt TrkB anterograde axonal transport, we studied the function of CC1-EGFP on the interaction between TrkB and kinesin-1 light chain 1 (KLC1), which has been reported to mediate TrkB anterograde transport bridged by JIP3.^16^ The results showed that CC1-EGFP significantly decreased the interaction between TrkB and KLC1 (Figures 2a and b). Then, we co-transfected TrkB-mRFP and CC1-EGFP into cultured hippocampal neurons and monitored the movement of TrkB-mRFP-containing vesicles using time-lapse fluorescence microscopy to further examine the effect of CC1-EGFP on the axonal anterograde transport of TrkB in live cells (Supplementary movies 1 and 2). The data showed that TrkB-mRFP-containing vesicles in the axons of CC1-EGFP overexpression neurons were more immobile than those in the EGFP overexpression neurons. Unsurprisingly, CC1-EGFP had no influence on the movement of TrkB in the dendrites (Figures 2c and d). The results indicated that CC1-EGFP specifically inhibited the anterograde axonal transport of TrkB. Next, we examined the density of TrkB immunostaining in the distal 30-*μ*m segments of the axonal tips was significantly decreased in both siJIP3 and CC1-EGFP-transfected groups compared with that in the EGFP group, suggesting that CC1-EGFP interfered with the localization of TrkB receptors at axonal tips (Figures 2e and f). Similarly, CC1-EGFP only reduced TrkB localization at the axonal tips but not at the dendritic tips, which further proved that CC1-EGFP only affected pre-synaptic TrkB location. To verify whether CC1-EGFP impaired the anterograde axonal transport of TrkB specifically, we studied the expression of JNK3, whose trafficking is also mediated by JIP3 in neurons.^17^ The results showed that the expression of JNK3 at axonal tips was significantly decreased when JIP3 was knocked down, which was in accordance with previous reports.^17^ In contrast, CC1-EGFP had no effect on JNK3 distribution at axonal tips (Figures 2g and h). Our results suggest that CC1-EGFP specifically blocks TrkB anterograde axonal transport.

### CC1-EGFP disabled BDNF-induced pre-synaptic vesicle release

**Figure 3:**
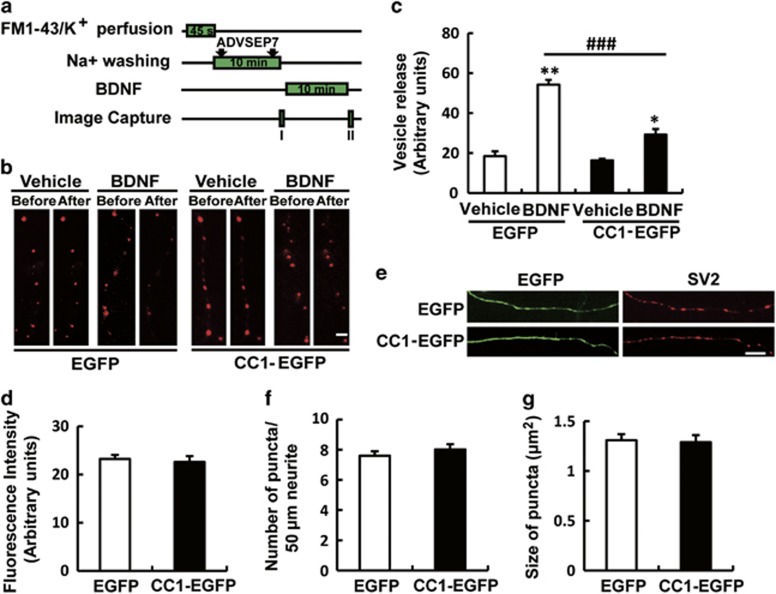
CC1-EGFP inhibited BDNF-induced pre-synaptic vesicle release in cultured hippocampal neurons. (**a**) Protocol for FM1-43 labeling and rinsing of pre-synaptic vesicles. Two images were captured before (I) and after (II) BDNF (50 ng/ml, 10 min) treatment. (**b**,**c**) CC1-EGFP inhibited BDNF-induced neurotransmitter release. Cultured hippocampal neurons (DIV7) were infected with an EGFP or CC1-EGFP-containing lentivirus. The FM1-43 assay was conducted in the neurons 48 h later according to (**a**). The releasable FM 1-43 fluorescence intensity (difference between images I and II) was quantitatively measured. Data are shown as the mean±S.E.M. from three independent experiments, >30 neurons per experiment (*n*=3; *P<0.05, **P<0.01 compared to the vehicle-treated EGFP control group; ^##^*P*<0.01 compared to the BDNF-treated EGFP group). Scale bar, 10 *μ*m. (**d**) Fluorescence intensity of FM1-43-positive puncta upon K^+^ stimulation. The results showed that CC1-EGFP did not change the number of synaptic vesicles in the active zone. Data of three independent experiments are shown and >30 neurons per experiment. (**e**–**g**) CC1-EGFP did not change the density and size of SV2-positive puncta in cultured hippocampal neurons. The numbers of neurons analyzed for each group was >30. Values are shown as the mean±S.E.M. from three independent experiments. Scale bar, 10 *μ*m

Pre-synaptic TrkB is important for BDNF-induced neurotransmitter vesicle release.^18^ Therefore, we determined to study whether CC1-EGFP affected it. An FM1-43 assay was performed and tested the exocytosis of synaptic vesicles induced by BDNF.^19^ The experimental protocol is shown in Figure 3a, and FM1-43 fluorescence intensity was measured by MetaMorph Software to quantify the release of the vesicles. Pictures were captured immediately before (stage I) and after (stage II) BDNF exposure and the gray intensity of each puncta was measured. The value of *I-II/I* represents the vesicle releasing ability at pre-synapses. The results showed that the value of *I-II/I* in the EGFP+BDNF group was greatly increased compared with that of the EGFP+Vehicle group, which suggested that our FM1-43 assay was effective. Importantly, CC1-EGFP significantly blocked the BDNF-mediated release of vesicles (Figures 3b and c), suggesting that CC1-EGFP disabled BDNF-induced neurotransmitter vesicle release. To exclude the possibility that CC1-EGFP disabled the vesicle release by downregulating the FM1-43 uptake ability in the active zone, we measured the fluorescence intensity of FM1-43-positive puncta upon K^+^ stimulation (I stage). The results showed that CC1-EGFP did not alter the density of synaptic vesicles (Figure 3d), indicating that CC1-EGFP blocked the function of BDNF to enhance the release of pre-synaptic vesicles. Meanwhile, to eliminate the possibility that CC1-EGFP influenced neurotransmitter vesicle recruitment at the pre-synapse, we examined the immunostaining levels of SV2-positive puncta. The results showed that CC1-EGFP did not change the number and size of SV2-positive synaptic vesicles at axons compared with that in the EGFP group (Figures 3e–g). These results indicated that CC1-EGFP specifically reduced BDNF-mediated pre-synaptic vesicle release rather than recruited the particles at the active zone.

### CC1-EGFP overexpression disrupted TrkB retrograde signaling

**Figure 4:**
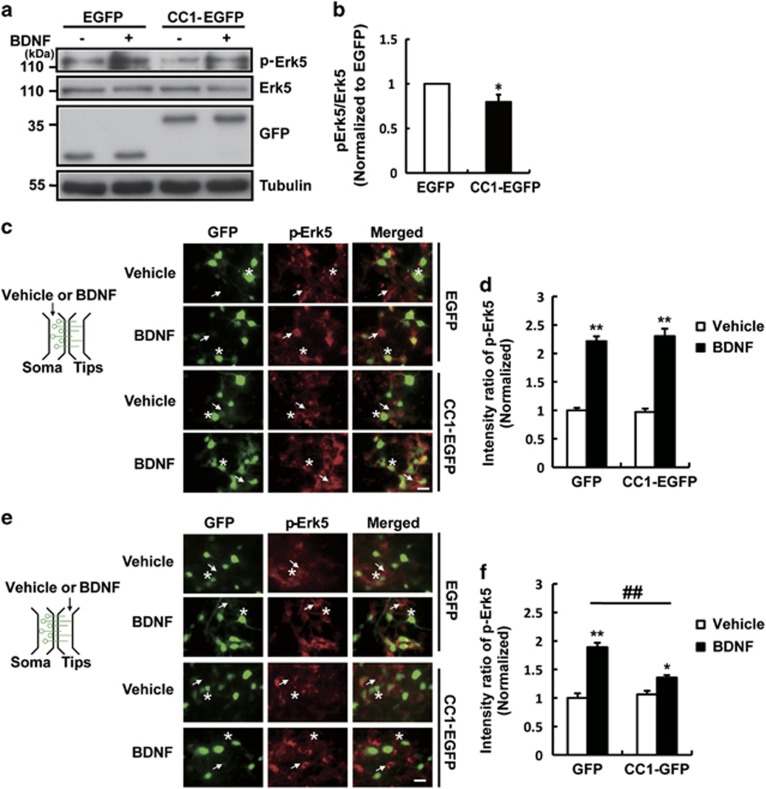
CC1-EGFP overexpression disrupted TrkB retrograde signaling. (**a**) Cultured hippocampal neurons (DIV7) were infected with GFP or CC1-EGFP contained lentivirus. 48 h later, neurons were starved for 8 h with serum-free medium and then were treated with BDNF (50 ng/ml) for 30 min. Cell lysates were collected and the level of Erk5 phosphorylation was detected by western blot. (**b**) Quantitative analysis of p-Erk5 level in (**a**). Data are shown as the mean±S.E.M. (*n*=3–4 per group, *P<0.05, compared with the EGFP group). (**c** and **e**) Hippocampal neurons infected with EGFP or CC1-EGFP constructs were cultured in the microfluidic chambers for 6 days. BDNF (50 ng/ml) or control vehicle was applied to the cell body compartment (**c**) or the axonal compartment (**e**) for 30 min. Then, cells were stained with anti-GFP (green) and anti-pErk5 (red) antibodies. Images of cell bodies are shown. Asterisk indicates EGFP or CC1-EGFP-positive neurons, and arrow indicates the uninfected wild-type neurons. Scale bar, 10 *μ*m. (**d** and **f**) Quantitative analysis of the intensity of p-Erk5 in the cell bodies after BDNF treatment to the cell body compartment (**d**) or axonal compartment (**f**). The numbers of neurons analyzed for each group was >30. Values are shown as the mean±S.E.M. (*n*=3, *P<0.05, **P<0.01 compared to the vehicle-treated EGFP group; ^##^*P*<0.01 compared to the BDNF-treated EGFP group)

Neurotrophins have been reported to be secreted into the synaptic cleft and reabsorbed by pre-synaptic Trk receptors. The activated Trk signal is retrogradely transmitted through the axon to the soma to elicit the function of BDNF.^20^ The retrograde signaling of BDNF-TrkB has been proven to activate extracellular signal-related protein kinase 5 (Erk5) and advance neuronal survival.^21^ Therefore, we measured the activation levels of Erk5 to represent the effect of CC1-EGFP on BDNF-induced retrograde signaling transmission. The results showed that CC1-EGFP inhibited BDNF-triggered phosphorylation of Erk5 in cultured hippocampal neurons (Figures 4a and b). To further address the role of CC1-EGFP on pre-synaptic TrkB, we employed a microfluidic chamber that contained a 450-*μ*m groove connecting two parallel compartments to separate the cell bodies and axons of cultured hippocampal neurons, and the cell bodies or axons could be individually treated. Then, we examined BDNF-induced Erk5 activation by immunostaining. The results showed that BDNF treatment at the cell body increased Erk5 phosphorylation in the cell bodies, and CC1-EGFP had no effect on BDNF-induced phosphorylation of Erk5 in the cell bodies (Figures 4c and d). These suggested that CC1-EGFP did not affect the signaling transmission which originated from the cell bodies or the dendrites. However, when BDNF exposed to the axonal tips, we found that BDNF stimulation at the distal axons led to rapid phosphorylation of Erk5 in the cell bodies. More importantly, CC1-EGFP decreased BDNF-induced Erk5 activation compared with that in the EGFP group (Figures 4e and f). These data suggested overexpression of CC1-EGFP impaired BDNF-induced axonal retrograde signaling. Overall, the above *in vitro* experiments reflected that CC1-EGFP interrupted TrkB distribution at the axonal tips by inhibiting the formation of the TrkB axonal anterograde trafficking complex, impaired BDNF-induced neurotransmitter release and retrograde signaling.

### Pre-synaptic TrkB in BLA neurons involved in memory extinction

**Figure 5:**
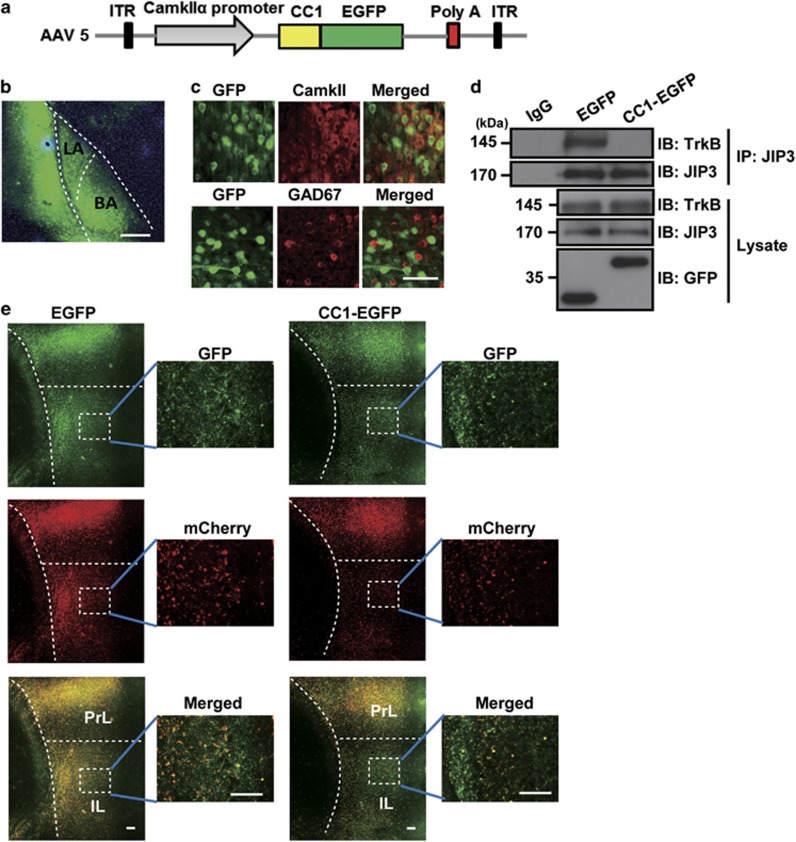
CC1-EGFP overexpression in BLA neurons. (**a**) Construction of the CamkII*α* promoter-driven CC1-EGFP in an AAV5 virus vector. (**b**) The localization and diffusion scope of the AAV5 virus in the BLA; scale bar, 100 *μ*m. (**c**) CC1-EGFP was predominately expressed in CamkII*α*-positive cells and rarely expressed in GAD67-positive cells; scale bar, 50 *μ*m. (**d**) CC1-EGFP interrupted the endogenous interaction of JIP3 with TrkB. BLA neurons infected with EGFP or CC1-EGFP-containing AAV5. After that, BLA tissue were lysed and subjected to immunoprecipitation with anti-JIP3 antibody. TrkB, JIP3 and GFP were detected by immunoblotting. The figure represents three independent experiments that yield similar result. (**e**) CC1-EGFP decreased the distribution of TrkB at the axonal tips of BLA neurons. Rats BLA region were injected with AAV5-CKII-TrkB-mCherry which mixed with AAV5-CKII-EGFP or AAV5-CKII-CC1-EGFP. Green staining was the terminal of BLA neurons, and red represented exogenous TrkB. Pictures showed the IL region of rats. Scale bar, 10 *μ*m. (*n*=4 per group)

Previous study has shown the TrkB receptor in the BLA plays a necessary role in memory extinction, and overexpression of TrkB.T1 in the BLA impaired memory extinction.^12^ However, whether pre- or post-synaptic TrkB in the BLA neurons is responsible for the long-projection circuit for fear memory extinction remains uncertain. Firstly, the distribution of TrkB in amygdalar neurons were tested by immunostaining, and CC1-EGFP attenuated the number of axonal TrkB (Figures S1A–B). To explore the function of pre-synaptic TrkB *in vivo*, we created an AAV5 virus that carried EGFP or CC1-EGFP under the regulation of the CamkII*α* promoter (Figure 5a), which only drives the protein expression in long-projection excitatory neurons. Then, the AAV5 viruses were microinjected into the BLA region, which was confirmed by immunofluorescence staining (Figure 5b). To determine the neuronal population expressing CC1-EGFP, we detected the expression of EGFP in the BLA region by immunohistochemistry. The immunohistochemical analysis showed that CC1-EGFP was mainly expressed in CamkII*α*-positive excitatory projection neurons, while it was rarely detected in GAD67-positive GABAergic interneurons (Figure 5c). This result was consistent with previous work and proved that CamkII*α* promoter could regulate the expression of CC1-EGFP as expected.^22^ Next, we examined the function of CC1-EGFP on the TrkB anterograde trafficking from BLA to IL. Firstly, we performed an endogenous TrkB/JIP3 co-immunoprecipitation assay in EGFP or CC1-EGFP-injected BLA to prove whether CC1-EGFP interfering the association of TrkB with JIP3 in BLA neurons *in vivo*. The results showed that CC1-EGFP significantly inhibited the interaction of TrkB with JIP3 in BLA neurons (Figure 5d) and suggested CC1-EGFP might regulate TrkB anterograde transport in BLA neurons *in vivo*, which is similar to the results Figure 1e. Secondly, we investigated whether the presynaptic TrkB at IL projected from the BLA neurons was downregulated. An AAV5 virus expressing mCherry tagged TrkB (AAV5-CKII-TrkB-mCherry) was constructed. The mixture of AAV5-CKII-TrkB-mCherry with AAV5-CKII-EGFP or AAV5-CKII-CC1-EGFP were injected in the BLA. As shown in Figure 5e, and we found CC1-EGFP led to significant reduction of axonal TrkB at IL projected from BLA neurons. All of the results suggested that CC1-EGFP could be applied to inhibit TrkB anterograde transport and to study the function of pre-synaptic TrkB *in vivo*.

**Figure 6:**
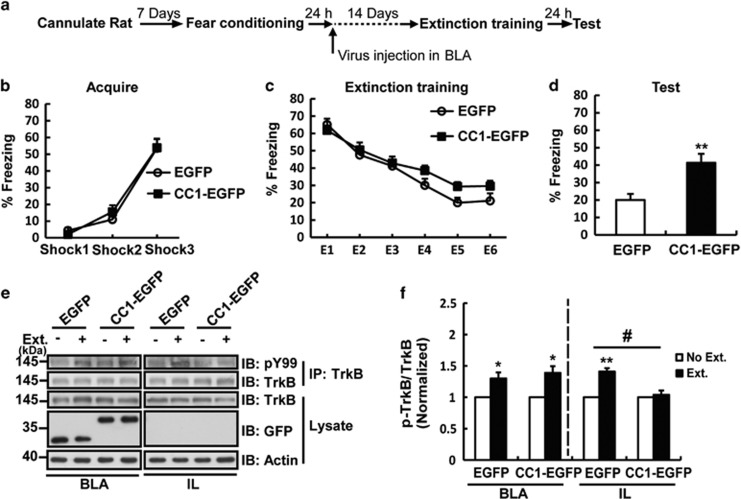
Expression of CC1-EGFP in the BLA impaired the extinction of fear memory. (**a**) Schematic diagram of the behavioral protocol. Rats received bilateral microinjection of EGFP or CC1-EGFP-containing AAV5 virus 24 h after cued fear conditioning training, and extinction training was performed 14 days later. (**b**–**d**) Freezing responses of rats during fear memory acquisition training, extinction training and testing. Data are shown as the mean±S.E.M. (EGFP group *n*=8, CC1-EGFP group *n*=9, *P<0.05, **P<0.01 compared to the EGFP group). (**e**,**f**) Phosphorylation levels of TrkB in the BLA and IL 2 h after extinction training. Lysates of the BLA and IL were subjected to immunoprecipitation with the anti-TrkB antibody and were immunoblotted with the pY99 antibody. Densitometric analysis showed the relative levels of p-TrkB compared to total TrkB from the immunoprecipitation. Data are shown as the mean±S.E.M. (*n*=3 per group, *P<0.05, **P<0.01 compared to the EGFP-infected no extinction group; ^#^*P*<0.05, compared to the EGFP-infected extinction group)

Next, the effects of CC1-EGFP on cued fear memory extinction was investigated. AAV5-CKII-EGFP or AAV5-CKII-CC1-EGFP was injected into the BLA before conditioned fear training. Surprisingly, CC1-EGFP impaired long-term memory (Supplementary Figures S2A–C). Therefore, we changed the time of the virus microinjection to 24 h after conditioned fear training when the fear memory had been consolidated. Fear memory extinction training was performed 14 days later, and the procedure is shown in Figure 6a. Compared with EGFP control, CC1-EGFP microinfusion did not alter cued memory acquisition and extinction training (Figures 6b and c); however, rats with CC1-EGFP injection showed a significantly higher freezing ratio during the extinction memory test period compared to EGFP injected rats (Figure 6d), indicating CC1-EGFP inhibited cued fear memory extinction. These results suggested that pre-synaptic TrkB of BLA projection neurons contributed to fear memory extinction.

### Pre-synaptic TrkB in BLA neurons mediated BDNF signaling transmission between the BLA and IL during memory extinction

A network of brain regions is responsible for cued memory extinction, including the connection between the BLA and central amygdala (CeA) as well as between the BLA and IL.^23, 24^ Previous work has demonstrated that BDNF signaling connection between the BLA and IL contributes to memory extinction.^25^ Therefore, we tested whether pre-synaptic TrkB was involved in the process of extinction and promoted the BDNF signaling transmission in BLA projection neurons. The results showed that CC1-EGFP overexpression in the BLA did not change the extinction-enhanced phosphorylation of TrkB in the BLA compared with that in the EGFP group 2 h after extinction training. Interestingly, CC1-EGFP blocked extinction-induced TrkB phosphorylation in the IL (Figures 6e and f). This result suggested that the CC1-EGFP-mediated blockage of memory extinction was caused by the decreased BDNF signal input to the IL during extinction training.

**Figure 7:**
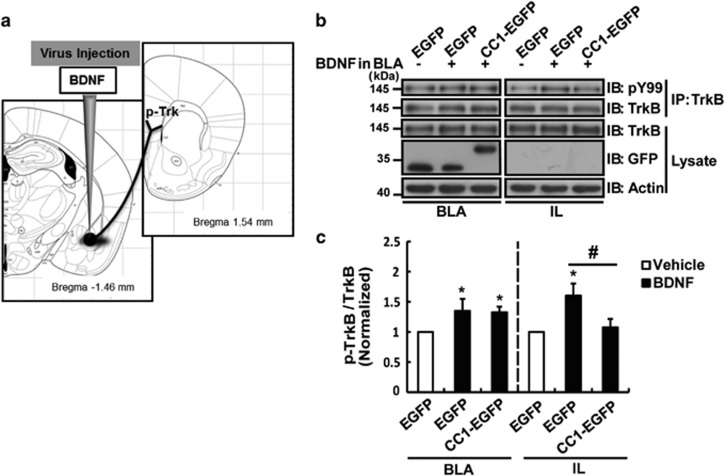
CC1-EGFP in BLA neurons inhibited mediated BDNF signaling transmission from the BLA to IL. (**a**) Graphic displaying the method of BDNF and virus injection. (**b**,**c**) Representative immunoblots and relative gray density analysis of p-TrkB. Microinjection of BDNF into the BLA induced a rapid increase in TrkB phosphorylation both in the BLA and IL within 20 min, and the increase in TrkB phosphorylation in the IL was blocked by CC1-EGFP expression in the BLA. Data are shown as the mean±S.E.M. (*n*=3 per group, *P<0.05 compared with the vehicle-treated EGFP group; ^#^*P*<0.05 in the IL compared with the BDNF-treated EGFP group)

Then, we verified whether the memory extinction impairment by CC1-EGFP was caused by the blockade of the transmission of the BDNF signal from the BLA to IL. We microinjected BDNF into the BLA and activated the local TrkB (Figure 7a). Specifically, 14 days after microinjecting the virus into the BLA, we administered exogenous BDNF into the BLA. Then, we examined the phosphorylation levels of TrkB in the BLA and IL 20 min later. The data showed that BDNF dramatically increased TrkB phosphorylation in both the BLA and IL in the EGFP+BDNF group. However, compared with that in the EGFP+BDNF group, overexpression of CC1-EGFP in the BLA inhibited the BDNF-induced enhancement of TrkB phosphorylation in the IL but not the BLA (Figures 7b and c). These results suggested that CC1-EGFP inhibited BDNF signaling transmitted from the BLA to the IL, and the fear memory extinction may rely on the function of pre-synaptic TrkB in the BLA neurons.

## Discussion

Our study demonstrated that CC1-EGFP inhibited TrkB anterograde axonal transport, caused less TrkB distribution in the pre-synapse and disrupted BDNF-induced pre-synaptic vesicle release and TrkB retrograde signaling transmission. Moreover, with the help of CC1-EGFP, we found that the blockage of pre-synaptic TrkB in BLA neurons impaired fear memory extinction by blocking anterograde BDNF signal transmission from the BLA to IL. Thus, we provided evidence that pre-synaptic TrkB in BLA neurons is involved in transmission of the BDNF signal from the BLA to IL.

First, we developed CC1-EGFP as an inhibitor of TrkB anterograde axonal transport, which could be applied to study the function of pre-synaptic TrkB *in vivo*. Studies with TrkB knockout mice and BLA region TrkB conditional knockout mice have shown memory formation impairment, suggesting that TrkB plays an essential role in memory behavior.^26, 27^ The TrkB receptor is widely distributed in axons, dendrites and soma. Researchers have primarily studied the function of pre-synaptic or post-synaptic TrkB in cultured neurons by overexpression of TrkB.T1 and electrophysiological recording *in vitro*.^28^ However, there isn’t direct evidence to support the function of pre- or post-synaptic TrkB in rat memory behavior. In this study, CC1-EGFP, as the anterograde-specific inhibitor of TrkB transport, impaired the distribution and function of pre-synaptic TrkB (Figures 1,2,3,4). More importantly, using CC1-EGFP, we clarified that pre-synaptic TrkB in BLA neurons mediated BDNF signaling transmission from the BLA to IL during memory extinction (Figures 5,6,7). With the help of CC1-EGFP, we provide a new way to study the functions of pre-synaptic TrkB *in vivo*, such as in brain development, LTP and BDNF-related mental disorders.

Second, our study revealed that pre-synaptic TrkB in BLA projection neurons is responsible for BDNF-mediated fear memory extinction. Neuronal inactivity or disruption of synaptic plasticity in the BLA is known to impair fear memory acquisition, retrieval and extinction.^3^ Furthermore, cellular plasticity in the BLA, which is mediated by BDNF and the NMDA receptor, is involved in the extinction memory process.^29^ These reports indicated that as a BDNF receptor, TrkB may be required for memory extinction. Not surprisingly, a study showed that TrkB.T1 overexpression in the BLA impaired startle memory consolidation and extinction, indicating that the TrkB receptor in the BLA is crucial for the integration and output of BDNF signaling during the memory process.^12, 30^ However, TrkB.T1 competes with the stimulation of pan-neuronal TrkB receptors, making it difficult to distinguish whether pre- or post-synaptic TrkB mediates BDNF signaling transmission during memory extinction. In this study, we demonstrated that pre-synaptic TrkB in BLA projection neurons is an important regulator responsible for fear memory extinction (Figures 6b–d,Supplementary Figure S2). Moreover, we discovered that pre-synaptic TrkB in BLA neurons mediated BDNF signaling transmission from the BLA to IL during fear memory extinction (Figures 6e and f). The results suggested that the BLA plays an important role in memory extinction process.

**Figure 8:**
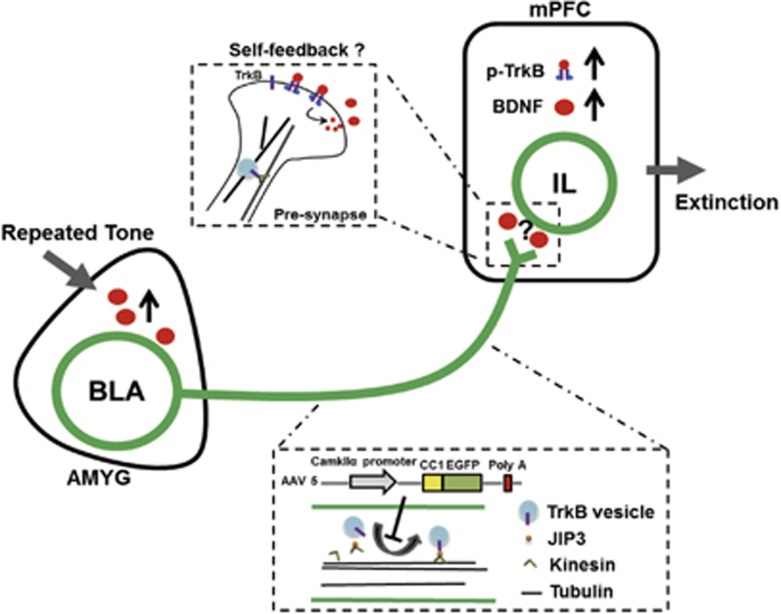
Work model. Pre-synaptic TrkB of BLA neurons mediated BDNF signaling transmission in fear memory extinction. Infusion of CC1-EGFP into the BLA region inhibits TrkB axonal anterograde transport and reduces its location at pre-synapse. Then, CC1-EGFP overexpression at the BLA impairs memory extinction by interrupting BDNF self-feedback system which depends on the pre-synaptic TrkB of BLA neurons

More importantly, our studies indicated that the BLA-IL neural circuit regulates memory extinction by pre-synaptic TrkB. Our previous work found that exogenous BDNF microinjection into the BLA could rapidly and strongly promote fear memory extinction, as well as TrkB phosphorylation in the IL,^25^ but it is still unclear how the BDNF signal is transmitted from the BLA to IL. BDNF is required for LTP, which depends on the pre-synaptic TrkB receptor.^31, 32^ Upon the release of BDNF, pre-synaptic TrkB is activated and gives rise to increasing BDNF release via a positive self-feedback mechanism,^33^ which explains BDNF signaling excitation of the IL is dependent on pre-synaptic TrkB in BLA projection neurons. Recently, an increasing number of studies have focused on the autocrine signaling transmission mode.^34^ Overall, we suggested that pre-synaptic TrkB-mediated BDNF signaling transmission from BLA to the IL is an element important for fear memory extinction (Figure 8). However, whether the BLA-IL circuit is sufficient for BDNF signaling-induced memory extinction is still unsolved. In addition to the BLA-IL circuit, researchers have found that hippocampal-infralimbic BDNF signaling is also involved in fear memory extinction,^6^ and the function of pre-synaptic TrkB in this circuit needs to be further investigated.

In conclusion, we developed an inhibitor CC1-EGFP, which can specifically disrupt TrkB anterograde axonal transport and TrkB distribution in the axonal tips. Furthermore, we demonstrated that CC1-EGFP could disturb pre-synaptic TrkB-mediated BDNF function on pre-synaptic neurotransmitter release and memory extinction in the BLA-IL circuit. Our researches provide a tool to investigate the function of pre-synaptic TrkB specifically, and bring us new insights into the mechanisms underlying fear memory extinction.

## Materials and methods

### Animals

Male Wistar rats (250–300 g) were individually maintained at 20 °C under a 12 h light/dark cycle. Water and food were available *ad libitum*. All procedures were in accordance with the *National Institutes of Health Guide for the care and use of laboratory animals* and were approved by the institutional animal care and use committee of Shandong University.

### Plasmid constructs

The Flag-rTrkB-FL, Myc-mJIP3, Myc-hKLC1 and TrkB-RFP constructs have been described previously.^13^ The CC1 domain (QLSGEQEVLKGELEAAKQAKVKLENRIKELEEELKRVKSEAVTARREPREEVEDVSSYLCTELDKIPMAQ) was obtained from Myc-mJIP3 by PCR and sub-cloned into the pEGFP-N1 vector. CC1-EGFP was separately cloned into the FUGW and pAAV-CKII-mCherry vectors for lentivirus and AAV5 virus packaging (gift from Prof. Hilmar Bading, University of Heidelberg, Germany). Rats siJIP3 target sequence has been referred before as the following: 5′-CAG GCC GAG GAGAAA UUC A-3′.

### Neuronal culture and transfection

Cultured hippocampal neurons were from embryonic day 18.5 (E18.5) Wistar rats. Hippocampi were removed from the embryos and digested with 0.05% trypsin/EDTA. Neurons were cultured in neurobasal medium (Invitrogen, Carlsbad, CA, USA) supplemented with 2% B27 and 0.5 mM glutamine. An incubator with saturated humidity, 5% CO_2_, and invariant temperature at 37 °C was utilized for cell culture. For immunofluorescence staining, coverslips coated with 0.1 mg/ml poly-D-lysine (Sigma, St. Louis, MO, USA) in six-well plates were used. Neurons were electroporated with plasmids in a Nucleofector device (Amaxa Biosystems, Cologne, Germany) according to the manufacturer’s instructions.

The methods for amygdalar neurons culture were based on a previous report.^35^ Briefly, Wistar rats pups at E18.5 were killed by exposure of CO_2_. Then, the brain was removed and the brainstems were sliced away. After that, the brain was placed ventral side up and a coronal cut was made anterior to the diencephalon and posterior to the optic chiasm. Along the lateral fissure, a diagonal cut was made and the amygdalar region was separated by peeling away from the cortex. The other cell culture processions are all the same as the upper hippocampal neuron culture instruction.

### Immunofluorescence analysis

Hippocampal neurons cultured for 3 days were fixed with 4% paraformaldehyde in PBS and permeabilized with 0.4% Triton X-100 in PBS. The samples were incubated with PBS containing 10% donkey serum for 1 h at room temperature and then were incubated with the primary antibodies: rabbit anti-GFP (1:500, Invitrogen), goat anti-TrkB (1:500, R&D, Minneapolis, MN, USA, AF1494), mouse anti-GFP (1:200, Millipore, Temecula, CA, USA, MAB3580), or rabbit anti-JNK3 (1:500, Millipore, 04-893) at 4 °C overnight. Samples were washed three times with PBS and incubated with fluorescent secondary antibodies conjugated to Alexa Fluor 488 or Alexa Fluor 594 (Invitrogen) for 1 h at room temperature. All of the images were captured with a Zeiss LSM780 confocal microscope fitted with a × 63 oil-immersion objective lens (Microstructural Platform of Shandong University).

### Co-immunoprecipitation assay

HEK293 cells were electroporated with plasmids in a Nucleofector device (Amaxa Biosystems, Cologne, Germany). Cells were then extracted 48 h later with TNE buffer containing 10 mM Tris, 150 mM NaCl, 1 mM EDTA, 1% NP-40, 10% glycerol and protease inhibitors. The cell lysates were precipitated with M2-Sepharose (Sigma) overnight at 4 °C. The beads were rinsed five times with the TNE buffer and boiled in sample buffer (Invitrogen) for SDS-PAGE. For investigating the endogenous interaction, DIV6 hippocampal neurons were infected with the control EGFP or CC1-EGFP lentivirus (2 × 10^9^ TU/ml 10 *μ*l per 10 m dish) and were lysed with TNE buffer 48 h later. Samples of lysates were incubated with mouse anti-JIP3 antibodies (1:100, Santa Cruz, Shanghai, China) or mouse IgG-Sepharose (Sigma) as a negative control. The immuno mix was precipitated using protein G-Sepharose beads (Sigma). The bound proteins were eluted and analyzed by immunoblotting with rabbit anti-JIP3 (1:1000, Santa Cruz), rabbit anti-TrkB (1:2000, Millipore) and rabbit anti-GFP (1:3000, Invitrogen) antibodies.

### Live cell imaging

The protocol for live cell imaging has been reported before.^13^ Hippocampal neurons were transfected with constructs in a Nucleofector device (Amaxa Biosystems) and were cultured in glass-bottomed 35-mm dishes (Willco Wells) for 3 days. The performance of neurons was obtained using an Eclipse TE 2000-U inverted fluorescence microscope (Nikon, Shanghai, China) and observed with a motorized Z drive using a × 63 oil-immersion objective lens (1.0 NA). Images of the cells selected for imaging were acquired every 1 s continuously for 1 min. The vesicle was handled and processed by the Meta-Morph software. A kymograph was generated by using NIH ImageJ.

### FM1-43 staining

The protocol was carried out as previously described.^36^ Lentivirus-infected hippocampal neurons were incubated with high K^+^-containing medium (31.5 mM NaCl, 90 mM KCl, 2 mM CaCl_2_, 2 mM MgCl_2_, 25 mM HEPES, 30 mM glucose) including 10 *μ*M FM1-43 (Invitrogen) for 45 s. The cultures were then washed with normal saline solution (119 mM NaCl, 2.5 mM KCl, 2 mM CaCl_2_, 2 mM MgCl_2_, 25 mM HEPES, 30 mM glucose) and 1 mM ADVASEP-7 (Santa Cruz, Shanghai, China) for 5 min. BDNF (50 ng/ml) was delivered for 10 min to quench the FM dye. Images were acquired both before and after stimulation I and II. To prevent network activity from promoting dye staining throughout the experiment, the AMPA receptor antagonist CNQX (Sigma, 20 *μ*M) and NMDA receptor antagonist D-2-aminophosphonovaleric acid D-APV (Sigma, 40 *μ*M) were added. The fluorescence intensity of individual boutons was measured with NIH ImageJ.

### SV2 staining and analyses

Lentivirus-infected neurons were fixed with 4% PFA on DIV9 and labeled with antibodies against the presynaptic protein SV2 (1:500, Developmental Studies Hybridoma Bank, Iowa City, Iowa, USA). Images were acquired with a × 63 oil-immersion lens. Threshold was set to twice the fluorescence intensity of the neuritis shaft devoid of synapses (background). The number and size of the SV2 puncta were determined by using the integrated morphometric analysis feature in NIH ImageJ.

### Neuronal culture in microfluidic chambers

The method for cell culturing in a microfluidic chamber has been previously described.^37^ Briefly, the microfluidic chambers were fixed to poly-D-lysine-coated 60-mm dishes. Suspensions of neurons were plated into the compartment of the cell soma; the other side was filled with neurobasal medium supplemented with B27. After 4 days of culturing, axons grew through the microgrooves and extended into the axonal compartment. Lentivirus was added into the cell body side, and cells were cultured for another 48 h. Then, BDNF (50 ng/ml) was administered in each compartment for 30 min. Cells were then fixed with 4% PFA and stained with mouse anti-GFP (1:500, Millipore) and rabbit anti-pErk5 (1:500, CST, Beverly, MA, USA) antibodies. Pictures were captured with a × 63 oil-immersion objective lens in the Eclipse TE 2000-U inverted fluorescence microscope (Nikon). The fluorescence intensity was measured by NIH ImageJ.

### Surgery and microinjection

Rats anesthetized with 5% chloral hydrate were implanted bilaterally with guide cannulas to the BLA or IL. The coordinates were as follows: BLA: anteroposterior (AP), −2.97 mm; lateral (L), ±5.1 mm; dorsoventral (V), −7.0 mm; IL: anteroposterior (AP), +3.14 mm; lateral (L), ±0.3 mm; dorsoventral (V), −4.0 mm. To prevent clogging, a stylus was placed into the cannula. Bilateral infusion cannulas (28 gauges) were inserted 7 days later, extending 1.5 mm beyond the tip of the guide cannulas. The injection cannula was linked via PE20 tubing to a 10-*μ*l Hamilton microsyringe motored by a microinjection pump (KDS 200, KD Scientific, Holliston, MA, USA). Infusions were administered with a volume of 0.5 *μ*l over the course of 2 min, and an additional 2 min was allowed for diffusion before the infusion cannulas were removed. BDNF (250 ng/*μ*l, 1 *μ*l/lateral) was administered into the BLA, and AAV5-CamkII*α*-EGFP (6.72 × 10^13^ vp/ml, ViGene Bioscience, Jinan, China, 1.5 *μ*l/lateral), AAV5-CamkII*α*-CC1-EGFP (5.84 × 10^13^ vp/ml, ViGene Bioscience, 1.5 *μ*l/lateral), and AAV5-CamkII*α*-TrkB-mCherry (1.64 × 10^14^ vp/ml, ViGene Bioscience, 1 *μ*l/lateral) were injected into the BLA 24 h after acquisition of the auditory fear memory.

### Cued fear conditioning and extinction

According to the previously reported protocol,^38^ the rats were placed into a standard fear conditioning chamber (Panlab, Barcelona, Spain). After 10 min of habituation without any stimulation, rats received three trials with 20 s, 5 kHz, 80 dB tones (CS), simultaneously ending with a 1 s, 0.75 mA foot shock (US). Each trial was separated by a 180-s inter-trial interval. After the last shock, the rats remained in the chamber for 60 s and were then placed back in their home cages.

On the next day, rats received an extinction training session that consisted of 19 CS exposures without a shock presentation. Each CS exposure was separated by a 180-s interval. After each session, the experimental chamber was cleaned with 70% alcohol. The rats received a test session consisting of four CS exposures 24 h later to assess the extinction of the cued fear memory.

### Immunohistochemistry

Rats were anesthetized with 5% chloral hydrate anesthesia (7.5 ml/kg, i.p.) and transcardially perfused with saline and 4% PFA. Brains were post-fixed in 4% PFA overnight and coronal sections were serially cut at 40 *μ*m on a Leica VT1200S vibratome. Immunohistological staining was performed on free-floating sections. Slices were blocked in a solution containing 0.4% Triton X-100 and 10% donkey serum and then stained with rabbit anti-GFP (1:500, Invitrogen), mouse anti-CamkII*α* (1:500, Thermo, Rockford, lL, USA) and mouse anti-GAD67 (1:2000, Millipore) antibodies before incubation with the appropriate fluorescence-conjugated secondary antibodies.

### Immunoprecipitation

BLA and IL brain tissue samples were removed and lysed with TNE buffer. The protein concentration of each sample was detected by the BCA reagent (Thermo). For analysis of TrkB phosphorylation (p-TrkB), 5 mg protein was utilized for immunoprecipitation with the rabbit anti-TrkB antibody (1:200, Millipore), followed by immunoblotting with anti-phospho-tyrosine pY99 (1:3000, Santa Cruz), goat anti-TrkB (1:2000, R&D) and rabbit anti-*β*-actin (1:1000, Sigma) antibodies. Ratios of p-TrkB/total TrkB derived from control groups were normalized to 1.0.

### Statistical analyses

Data were analyzed by Student’s *t*-test, one-way ANOVA or two-way ANOVA, followed by LSD *post hoc* comparisons. All values in the text and figures represent the mean±S.E.M., and *P*<0.05 was considered significant.
