# Supplementary material for: Pre-synaptic TrkB in basolateral amygdala neurons mediates BDNF signaling transmission in memory extinction
Source: Cell Death Dis. 2017 Jul 27;8(7):e2959–. doi: 10.1038/cddis.2017.302 (PMC5550851; doi:10.1038/cddis.2017.302)
Supplement: Supplementary Figure Legends [file cddis2017302x1.docx]

**Supplement figure legends:**

**Supplement 1: CC1-EGFP impaired TrkB anterograde transport in amygdalar neurons.**

(A-B) Cultured amgydalar neurons were electroporated with EGFP, siJIP3, or CC1-EGFP, and endogenous TrkB was stained with the anti-TrkB antibody (red) at DIV3. Scale bar, 10 μm. The intensity of TrkB at distal axons and dendrites was analyzed. Data are shown as the mean ± SEM from three independent experiments, > 60 neurons per experiment (n = 3; *p < 0.05; compared to EGFP group).

**Supplement 2: Expression of CC1-EGFP in BLA interrupted cued fear memory long term memory.**

(A) Schematic diagram showing the behavioral process. EGFP or CC1-EGFP contained AAV5 virus was injected into BLA. 14 days later, rats were conducted with cued fear conditioning training and were tested 24h later.

(B-C) Freezing responses of rats during fear memory acquisition training, long term memory testing (LTM). Data are shown as the mean ± SEM (EGFP group n = 8, CC1-EGFP group n = 9, *p < 0.05, **p < 0.01 compared to the EGFP group).
