# Supplementary figures and images for: Pre-synaptic TrkB in basolateral amygdala neurons mediates BDNF signaling transmission in memory extinction
Source: Cell Death Dis. 2017 Jul 27;8(7):e2959–. doi: 10.1038/cddis.2017.302 (PMC5550851; doi:10.1038/cddis.2017.302)

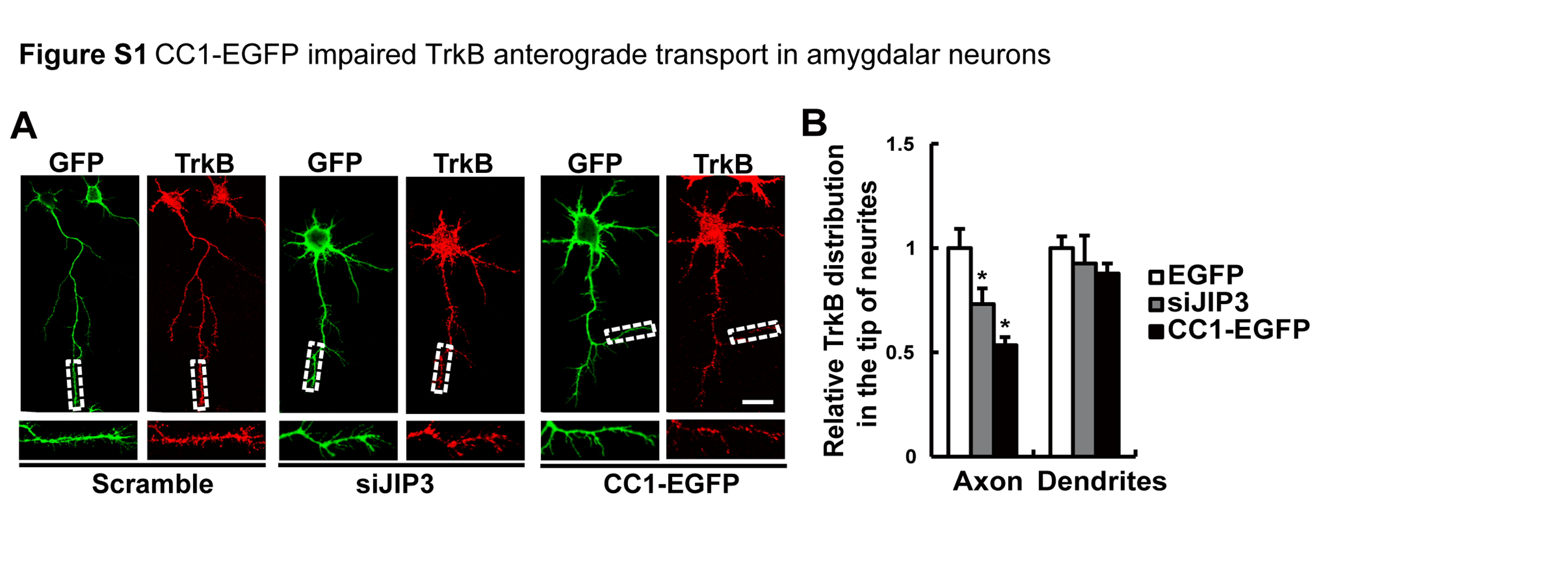

Supplement: Supplementary Figure S1 [file cddis2017302x2.tif]

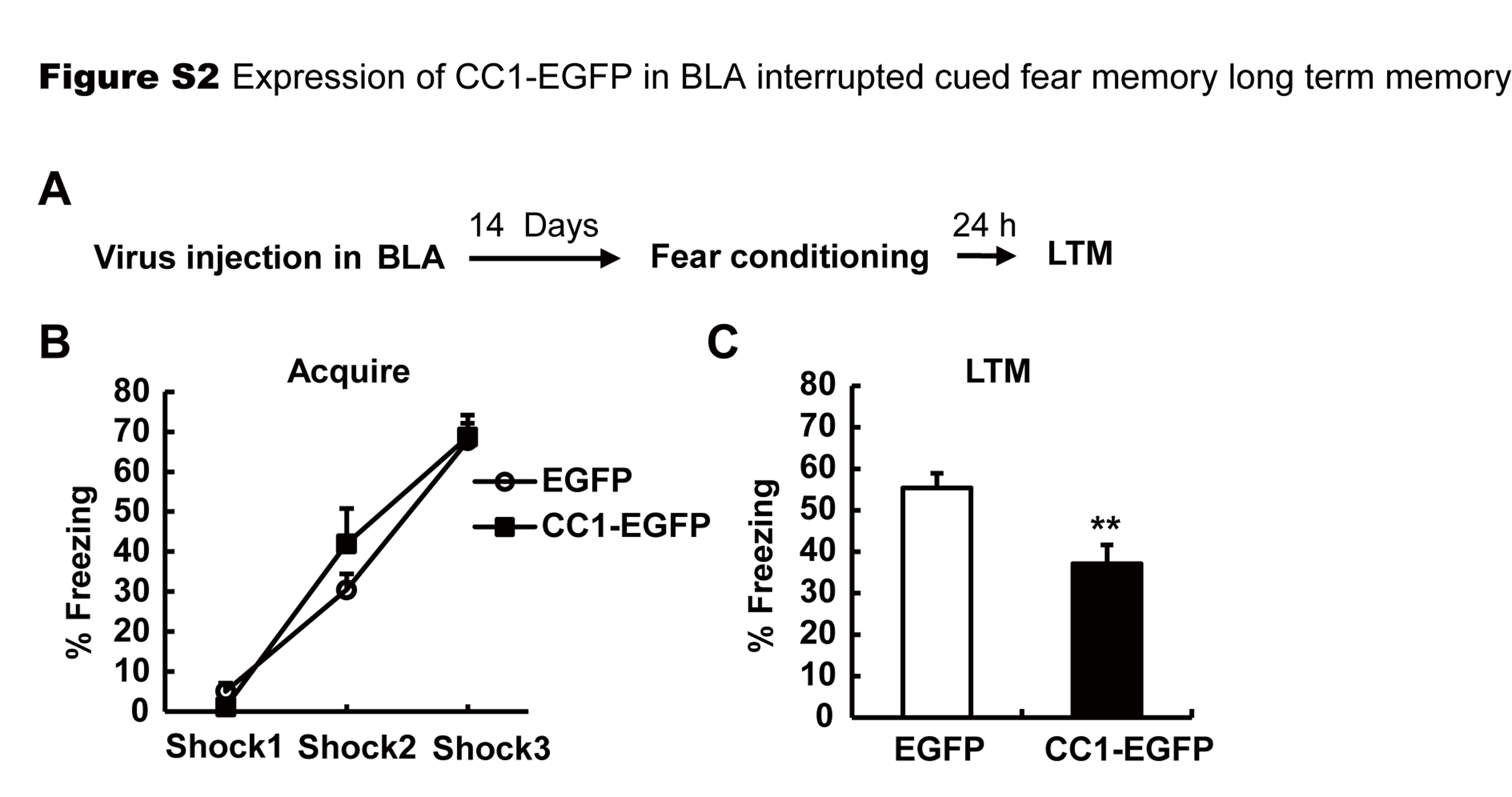

Supplement: Supplementary Figure S2 [file cddis2017302x3.tif]

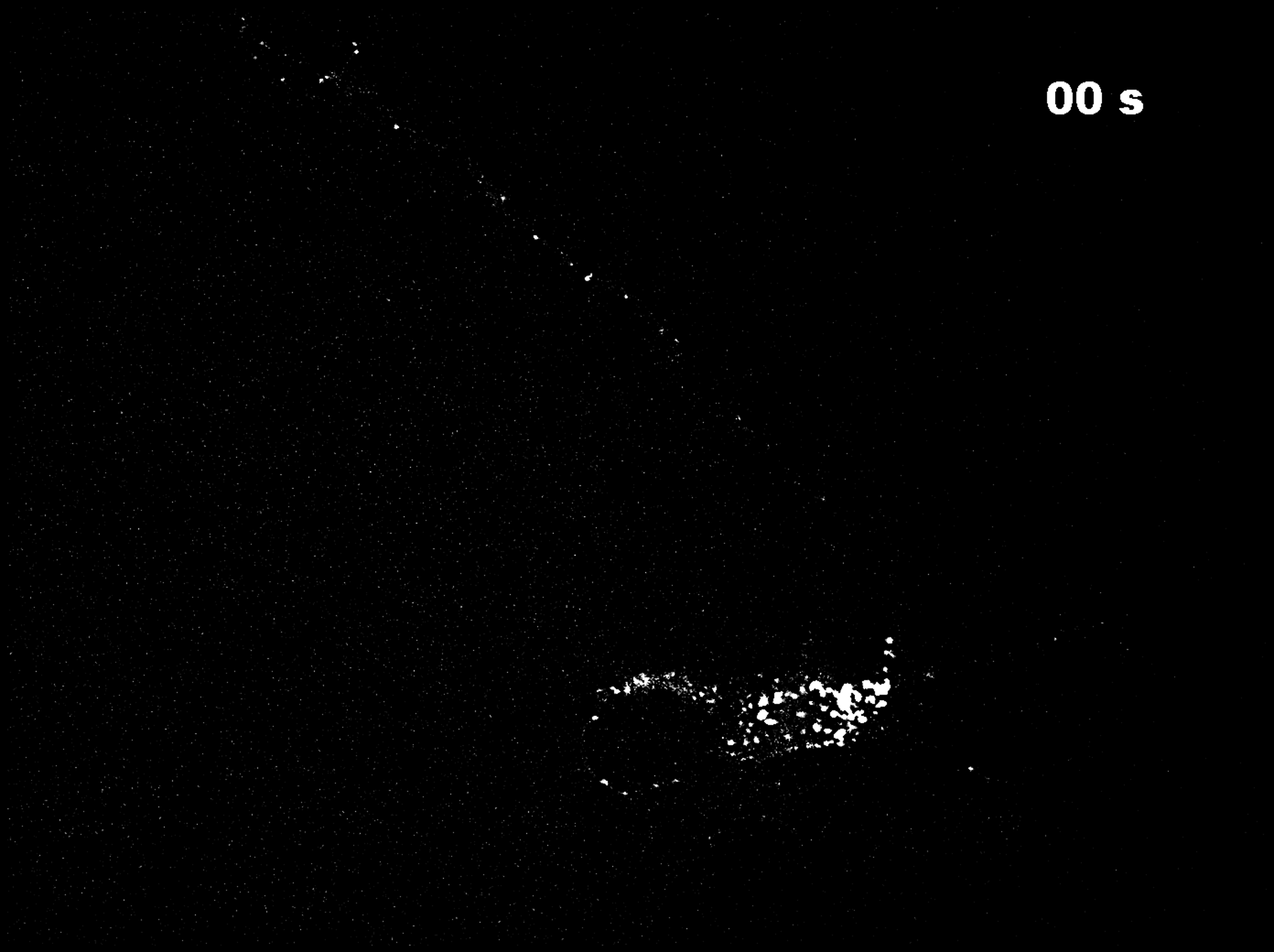

Supplement: Supplementary Figure S3 [file cddis2017302x4.tif]

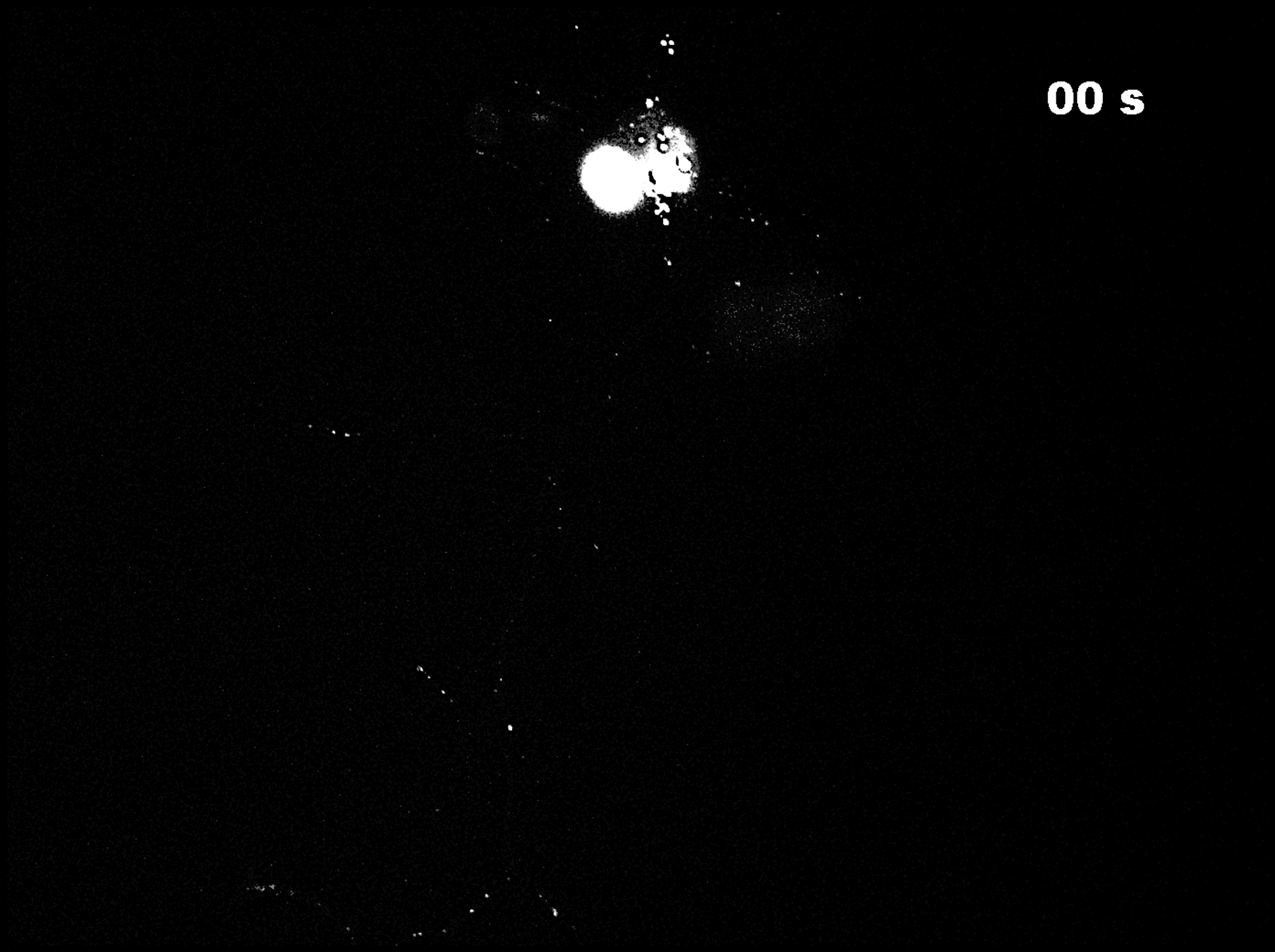

Supplement: Supplementary Figure S4 [file cddis2017302x5.tif]

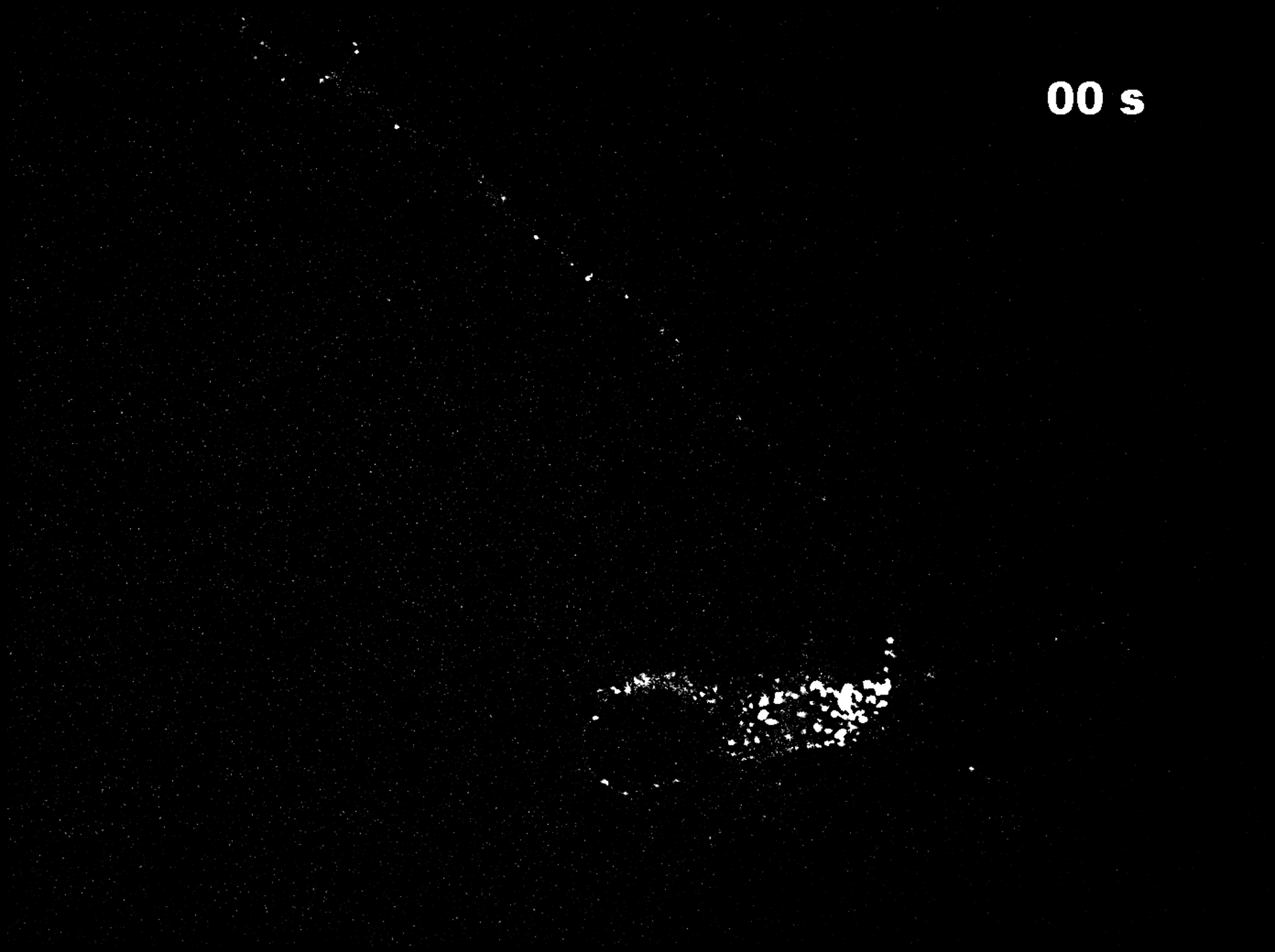

Supplement: Supplementary Movie S1 [file cddis2017302x6.tif]

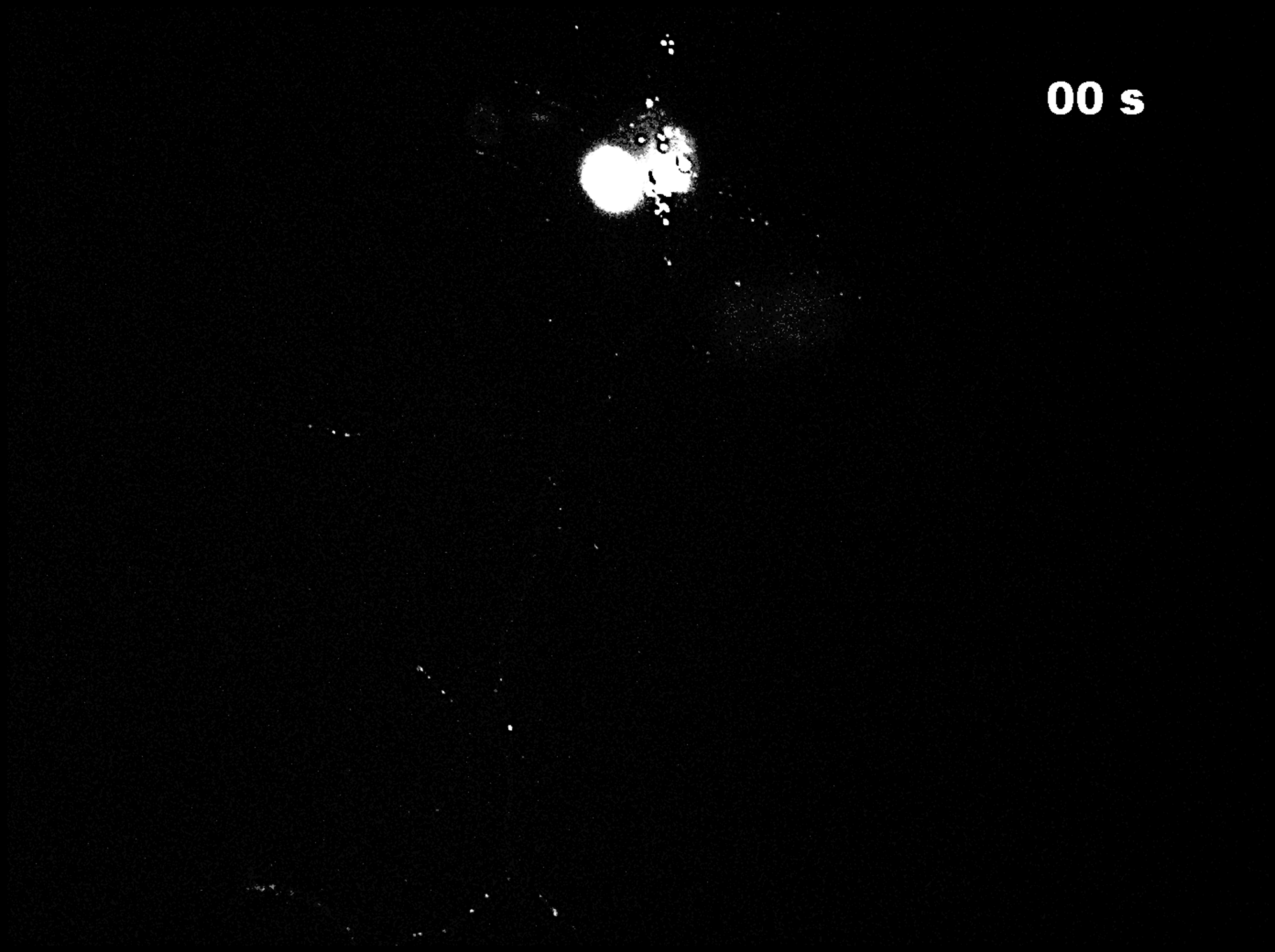

Supplement: Supplementary Movie S2 [file cddis2017302x7.tif]
