# Supplementary material for: Pre-synaptic TrkB in basolateral amygdala neurons mediates BDNF signaling transmission in memory extinction
Source: Cell Death Dis. 2017 Jul 27;8(7):e2959–. doi: 10.1038/cddis.2017.302 (PMC5550851; doi:10.1038/cddis.2017.302)
Supplement: Supplementary Movie Legends [file cddis2017302x8.docx]

**Supplementary Movie Legends**

**Movie 1**, Movement of TrkB-mRFP-containing vesicles in EGFP-transfected neurons. Hippocampal neurons were transfected with the EGFP and TrkB-mRFP constructs and the movement of TrkB-mRFP-containing vesicles were obtained using an Eclipse TE 2000-U inverted fluorescence microscope and observed with a motorized Z drive using a × 63 oil-immersion objective lens (1.0 NA).

**Movie 2**, Movement of TrkB-mRFP-containing vesicles in CC1-EGFP-transfected neurons. Hippocampal neurons were transfected with the CC1-EGFP and TrkB-mRFP constructs and the movement of TrkB-mRFP-containing vesicles were obtained using an Eclipse TE 2000-U inverted fluorescence microscope and observed with a motorized Z drive using a × 63 oil-immersion objective lens (1.0 NA).
